# To Subscribers

**Published:** 1893-10

**Authors:** 


### *EDITORIAL.*

The office of THE JOURNAL is in room 330 Equitable Building.

Address all communications, and make all remittances payable to THE ATLANTA MEDICAL AND SURGICAL JOURNAL, Atlanta, Ga.

Articles for publication should reach this office not later than the 15th instant.

The editors are not responsible for views expressed by contributors.

### TO SUBSCRIBERS.

You will receive this month your annual bill. You paid up pretty well in response to the last, for which we thank you.

Some of you neglected to pay, but you ought not, for every year makes the bill larger.

We are giving you the best two dollar journal in the country. It is worth paying two dollars for, and the man who pays nothing gets more than he is entitled to.

All who are in arrears will be reminded of it for a few months by seeing the date to which they have paid marked on THE JOURNAL wrapper.

The fellow who takes THE JOURNAL until he fears he may have to pay and then returns it marked "*refused*," stands a good chance of "getting his name in print." If everybody paid promptly much of the money would be spent for good reading matter. (We pay for some anyhow.)

Send money any safe way. THE JOURNAL expresses its kindest regards for you all.

*To those receiving sample copies: We will meet any proposition made you by any $2.00 journal.*

